# A systematic review of barriers to optimal outpatient specialist services for individuals with prevalent chronic diseases: what are the unique and common barriers experienced by patients in high income countries?

**DOI:** 10.1186/s12939-015-0179-6

**Published:** 2015-06-09

**Authors:** Elizabeth A. Fradgley, Christine L. Paul, Jamie Bryant

**Affiliations:** Priority Research Centre for Health Behaviour and Hunter Medical Research Institute, School of Medicine and Public Health, University of Newcastle, Callaghan, NSW 2305 Australia

**Keywords:** Health services, Outpatient, Cancer, Depression, Diabetes mellitus, Heart diseases, Joint diseases, Stroke, Systematic review, Accessibility

## Abstract

Health utilization and need assessment data suggest there is considerable variation in access to outpatient specialist care. However, it is unclear if the types of barriers experienced are specific to chronic disease groups or experienced universally. This systematic review provides a detailed summary of common and unique barriers experienced by chronic disease groups when accessing and receiving care, and a synthesized list of possible health service initiatives to improve equitable delivery of optimal care in high-income countries. Quantitative articles describing barriers to specialist outpatient services were retrieved from CINAHL, MEDLINE, Embase, and PyscINFO. To be eligible for review, studies: were published from 2002 to May 2014; included samples with cancer, diabetes mellitus, osteoporosis, arthritis, ischaemic heart disease, stroke, asthma, chronic pulmonary disorder (COPD) or depression; and, were conducted in high-income countries. Using a previously validated model of access (Penchansky and Thomas’ model of fit), barriers were grouped according to five overarching domains and defined in more detail using 33 medical subject headings. Results from reviewed articles, including the scope and frequency of reported barriers, are conceptualized using thematic analysis and framed as possible health service initiatives. A total of 3181 unique records were screened for eligibility, of which 74 studies were included in final analysis. The largest proportion of studies reported acceptability barriers (75.7 %), of which demographic disparities (44.6 %) were reported across all diseases. Other frequently reported barriers included inadequate need assessment (25.7 %), information provision (32.4 %), or health communication (20 %). Unique barriers were identified for oncology, mental health, and COPD samples. Based on the scope, frequency and measurement of reported barriers, eight key themes with associated implications for health services are presented. Examples include: common accommodation and accessibility barriers caused on service organization or physical structure, such as parking and appointment scheduling; common barriers created by poor coordination of care within the healthcare team; and unique barriers resulting from inadequate need assessment and referral practices. Consideration of barriers, across and within chronic diseases, suggests a number of specific initiatives are likely to improve the delivery of patient-centered care and increase equity in access to high-quality health services.

**Electronic supplementary material:**

The online version of this article (doi:10.1186/s12939-015-0179-6) contains supplementary material, which is available to authorized users.

## Introduction

In the last decade, chronic diseases such as cancer, heart disease and diabetes have become the leading cause of death worldwide and are associated with 59 % of deaths and 46 % of the global disease burden [1]. Chronic diseases are characterised by multiple causality, genetic and lifestyle risk factors, long latency periods, and prolonged periods of illness with some level of functional impairment or disability [2]. Individuals diagnosed with a chronic disease often suffer from reduced quality of life and report poor physical functioning and emotional wellbeing [3].

Individuals with chronic diseases are frequent users of complex and costly healthcare services [4]. Chronic disease care usually requires comprehensive and personalised services involving multi-disciplinary teams. This care is often delivered at outpatient clinics, which are defined as services providing diagnostic or therapeutic care not requiring an overnight stay in a medical institution [5]. Currently, non-emergency outpatient services for chronic diseases account for a large proportion of health expenditures within high-income countries [6]. In 2011, the Organization for Economic Co-operation and Development (OECD) estimated high income countries allocate, on average, approximately 33 % of their total healthcare budgets to outpatient services [6]. However, several countries dedicate an even larger proportion to these services including a variety of private and public-based systems.

With the associated high healthcare expenditure and disease burden, effective management of chronic diseases have been targeted in policy and research initiatives. Within high-income countries, emphasis has been placed on improving the efficiency and ability of health systems to respond to chronic disease patients’ evolving healthcare needs in an equitable manner. Several performance indicators relating to chronic care are incorporated into quality frameworks proposed by organizations such as the Institute of Medicine [7], the Australian National Health Performance Committee [8], the United Kingdom’s National Institute of Health [9], and the World Health Organization [10]. Suggested performance domains focus on equity, effectiveness, safety, responsiveness, continuity of care, efficiency and accessibility. Beyond these domains, patient-centered care is also considered to be essential to high quality healthcare and requires patients’ preferences and values to be considered in healthcare provision [11].

Accessibility is defined as the ability to receive timely resources to manage personal healthcare needs in order to achieve the best possible outcomes [12]. Several theoretical frameworks have been proposed in order to differentiate and operationalize the factors that can act as potential barriers to receiving care [13]. Roy Penchansky and William Thomas suggested a model of fit where access is conceptualized as the degree of fit between patient need and the service’s ability to respond to and meet those needs [14]. Poor ‘fit’ will result in an access barrier. Five distinct forms of barriers have been proposed and validated within this model (Table 1). Metrics used to describe these potential barriers to service access have included: 1) equitable patterns of service utilization according to demographic, clinical, or health insurance characteristics; 2) having a usual source of care; 3) patient need assessment, for example levels of unmet medical, supportive care, or prescription needs; and 4) patient satisfaction surveys [12, 15–17].

**Table 1:** Definition of barriers within the model of fit

Form of barrier	Definitions [107]
Availability	The relationship between the volume or type of existing services and patient volume or type of needs.
Accessibility	The relationship between the location of health services and the location of the patients.
Accommodation	The relationship between the manner in which the supply resources are organized to accept patients and the patients’ ability to accommodate to these factors.
Affordability	The relationship between prices of services and the patients’ ability and willingness to pay for these services.
Acceptability	The relationship between patients’ attitudes to personal and practice characteristics of existing providers and alternatively, provider perceptions of patients’ characteristics.

There is considerable inequity in access to high quality outpatient services. Health service utilization data has consistently demonstrated an association between patient characteristics and access barriers for individuals with chronic diseases. For example, ethnic minorities within the United States have been found to be significantly less likely to access outpatient services for asthma, hypertension, diabetes mellitus or congestive heart failure as compared to Caucasians [18]. This trend has also been identified in access to oncology services [19].

The proportion of unmet needs reported by patients is significantly higher for those with chronic diseases and increases with comorbidities [20]. Results from the Canadian Community Health Survey and national hospitalisation data report that unmet needs in samples of people with chronic diseases remain disproportionally high even after controlling for socio-demographic characteristics [20]. Research also suggests individuals with chronic diseases (lasting at least 6 months with restrictions in activities of daily living) were three times more likely to report an unmet need than individuals without a chronic disease [4]. Overall, health service utilization and need assessment survey data suggest individuals with chronic diseases struggle to access required health services; while these health services struggle to meet patients’ ongoing needs.

Health service planning and policy would benefit from detailed information on the scope of common and unique (i.e., disease-specific) barriers to optimal care. Currently, there is a lack of research comparing the barriers to care experienced across groups with chronic diseases [21]. While there are some trends in the types of barriers experienced by these groups, there has been no overarching review to distinguish experiences or concerns which are common across chronic disease groups compared to those which are unique to particular groups or diseases. Understanding the unique barriers to care experienced by particular groups may help to guide health service research to develop quality initiatives to target specific accessibility issue; conversely, those barriers that are common across groups should be prioritised and managed on a system-level.

This systematic literature review will examine the common and unique barriers experienced by nine chronic disease groups when accessing specialist outpatient care. For the purposes of this review, the definition of barrier proposed within the model of fit will be used - any factor which impedes or reduces the availability, accessibility, affordability, accommodation or amenability of outpatient care [14]. Additional factors that influence patient unmet needs, utilization patterns, and satisfaction that are not adequately captured by the model of fit will also be recorded. This includes patient-centered care domains, such as support for self-management or care coordination within multidisciplinary teams, that have recently become corner-stones of healthcare quality initiatives [11, 22]. The results will be highly applicable to a range of chronic disease health services and will be the preliminary step to understanding how limited access and unmet needs can be appropriately addressed by quality improvement initiatives within specialized outpatient settings.

### Objectives

This systematic review of quantitative studies was conducted to describe:

1. The scope and frequency of barriers reported by chronic disease patients when accessing outpatient specialist services;
2. The common and unique barriers that are reported across or within chronic diseases.

Beyond providing a quantitative description of the scope, frequency, and commonality of barriers experienced when accessing services, recurrent themes within the reviewed studies were summarized and framed within the context of health service interventions. This synthesis of study results provides a preliminary understanding of those approaches capable of improving the equitable delivery of chronic disease outpatient care within high-income countries.

## Review

### Methods

A systematic literature review of quantitative studies was conducted according to The Preferred Reporting Items for Systematic Reviews and Meta-Analyses (PRISMA) statement [23].

### Search strategy

Search terms were generated iteratively by the research team and reviewed by an experienced medical librarian. Search terms used in various combinations included: chronic disease; neoplasm; outpatient or ambulatory services. The following search limits were applied: English language; all adults defined as over the age of eighteen years; and publication date between 2002 and 2014. This year range was applied to capture articles published in response to several seminal articles released in 2001 that proposed accessibility as a quality indicator. This includes the Institute of Medicine’s Crossing the Quality Chasm [7]. An example of the electronic search strategy is available in the Supplementary Material (Additional file 1).

### Information sources

The search was conducted in: the Cumulative Index to Nursing and Allied Health Literature (CINAHL); Embase; MEDLINE; and PsychINFO. The final search was completed May 2014.

### Eligibility criteria

Quantitative or mixed methods studies which report barriers to receiving optimal specialist outpatient care were eligible for review. Six inclusion and eight exclusion criteria were applied to retrieved articles (Table 2). To ensure articles were relevant within high-income countries, only research conducted in 31 high-income Organization for Economic Co-operation and Development (OECD) countries were eligible for review [24]. A total of nine prevalent chronic diseases were included: Type 2 diabetes, arthritis, osteoporosis, ischaemic heart disease (coronary heart disease), stroke, depression, asthma, non-melanoma cancers, and chronic obstructive pulmonary disorders. These diseases were selected as they have been proposed as health priority areas within Australia [25], the Pan-Americas [26], Europe [27], and are included in major WHO reports relating to chronic diseases [28].

**Table 2:** Eligibility criteria for all retrieved articles

Inclusion criteria	Exclusion criteria
1. Quantitative or mixed methods study design	1. Qualitative study design, editorial letters, opinion articles or teaching documents
2. Adult patient, health service professionals or support persons are sampled	2. Paediatric samples (less than 18 years of age)
3. Study setting is an outpatient specialist service	3a. Participants are recruited from outpatient settings, but barriers to other care settings are assessed
	3b. Palliative, emergency or in-patient services only
	3c. Non specialist services only (such as primary care practices)
4. Study must clearly specify one or more of diseases of interest are included in the study sample.	4. Acute or other chronic diseases not listed as diseases of interest
5. A barrier to optimal outpatient care is measured	5. No barrier is measured (eg. treatment efficacy, diagnostic protocol, symptom or disease prevalence)
6. High income OECD countries^a^	6. All middle or low income non-OECD countries
7. Full text articles published in English	7. Conference proceedings, unavailable full text articles or article not published in English

^a^Defined by the World Bank based on 2011 Gross National Income per capita [24]

Paediatric research was excluded. Research involving childhood cancer survivors was included if the majority (>50 %) of participants were eighteen years of age or older. Several studies explored barriers across specialist, primary care, and inpatient services – these studies were only included if the majority of participants (>50 %) accessed outpatient services or a sub-group analysis was performed. Eligibility criteria were independently pilot tested by two members of the research team with a random sample of titles and abstracts (10 %).

### Study selection process

Using the eligibility criteria, a research team member reviewed all titles and abstracts. A random 10 % of these were reviewed by an independent secondary reviewer. A Cohen’s kappa value was recorded to assess inter-rater reliability. Discrepancies between the two reviewers were discussed, and if unresolved, a third reviewer was included to reach consensus. The study selection process was facilitated by Synthesis, a literature review software package [29].

### Data collection process

Study characteristics and data describing the barriers to receiving optimal outpatient care were extracted from full-text articles using a structured electronic form. All eligible full-text articles were coded by one reviewer, with a random 10 % of articles coded by a second independent reviewer. Coded results from the two reviewers were compared to ensure the process was systematic and comprehensive.

### Data items

Data items were extracted to address the following study objectives:

Objective 1: To describe the scope and frequency of barriers experienced when accessing specialist outpatient services, the following was recorded: 1) if a barrier relating to one of five domains within the model of fit - availability, accessibility, affordability, accommodation or acceptability (defined in Table 1)- was assessed; 2) the disease(s) of interest; and 3) the service(s) of interest. To describe any additional variables focusing on any barriers to optimal outpatient care that were not adequately captured within the model of fit, patient-centered care domains including information provision, self-management, need assessment, coordination of care, and medical errors were also recorded.

For each of the five domains defined in the model of fit and for additional barriers to optimal care, key terms were used to describe barriers in more detail. Where possible, Medical Subject Headings (MeSH) were chosen. For example, a general affordability barrier could be described as inadequate insurance coverage (MeSH: health insurance) or inability to pay for initial services or ongoing care (MeSH: medical fees).

Objective 2: To describe the common and unique barriers reported by chronic diseases, the number of disease groups reporting the barrier was recorded. A barrier was considered common if reported in relation to three or more diseases. Alternatively, a barrier was considered unique if reported in relation to one or two diseases. This range was selected as the high volume of oncology studies masked potential unique barriers experienced by only one other chronic disease, such as depression.

Finally, in order to frame these results within the context of health service interventions, the research team summarized emerging concepts using a thematic analysis approach [30]. To determine those concepts which were of most significance and relevance to outpatient service, raw study data were recorded and recurrent themes were summarized by the research team. This is considered as a data-driven thematic approach [30].

### Summary measures

If reported, the proportion or odds ratio of participants indicating a barrier was recorded as raw data. Due to the heterogeneity of study designs and outcome measures, meta-analysis could not be conducted.

## Results and discussion

### Study selection

**Fig. 1:**
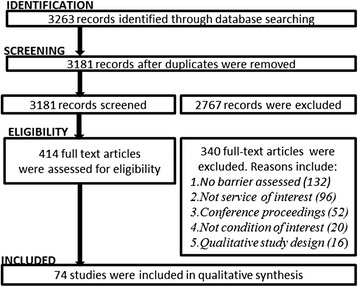
Study selection and screening process

A total of 3263 records were identified using the electronic search strategy, of which 3181 were unique records (Fig. 1). The eligibility screening process excluded 2767 abstracts. The initial kappa value reported for agreement between the two raters when reviewing the first 10 % of abstracts (selected using a statistical software random number generator) was 0.72, indicating substantial inter-rater reliability [31]. After discussion, all eligibility disagreements were resolved.

A total of 414 full text articles were screened for eligibility. The initial kappa value reported for agreement between the two raters when reviewing the first 10 % of full text articles (selected using a statistical software random number generator) was 1.0, indicating perfect agreement [31]. The eligibility screening process excluded 340 full text articles. The most common reasons for exclusion were not including a specialist outpatient setting (28.2 %), not measuring any barriers (38.8 %), or conference proceedings (15.3 %). One paper was excluded as the authors did not respond to a request for additional clarification on the applied study measure. A total of 74 articles met eligibility criteria and were included in the review (Fig. 1).

### Study characteristics

The majority of studies employed a descriptive cross-sectional survey design (50 of 74 studies, 67.6 %) [32–81] and all chronic diseases of interest were reported in at least one article. However, the volume of articles differed between chronic diseases: 59 articles (79.7 %) included oncology samples [19, 32–40, 42–47, 50–55, 57–63, 65–72, 74, 76–96]; 12 articles (16.2 %) included depression [32, 33, 41, 62, 64, 75, 83, 93, 97–100]; 10 articles (13.5 %) included diabetes [18, 32, 33, 64, 74, 75, 83, 101–103]; 10 articles (13.5 %) included ischaemic heart disease [18, 32, 33, 48, 56, 64, 74, 75, 83, 102]; 7 articles (9.5 %) included COPD [32, 33, 49, 56, 74, 75, 102]; 7 articles (9.5 %) included asthma [18, 32, 33, 64, 74, 75, 83]; 6 articles (8.1 %) included arthritis [32, 33, 73–75, 83]; and 3 articles (4.1 %) included osteoporosis [73–75]. A total of 12 studies (16.2 %) included more than one disease of interest [18, 32, 33, 56, 62, 64, 73–75, 83, 93, 102]. As such, excepting oncology, diseases of interest were predominately analysed as part of a cluster.

## Results of individual studies

### Objective 1: The scope and frequency of accessibility barriers

On average, studies examined 1.67 (SD = 1.11) of the five overarching barriers to specialist care outlined in the model of fit. No study examined all five overarching barriers. The scope and frequency of barriers reported for each domain is provided in Table 3.

**Table 3:** Percentage of reviewed studies reporting each overarching and specific barrier to specialist outpatient care (n = 74)

Barrier to outpatient services		Percentage of studies (n)	References
Availability		28.4 (21)	[32–34, 37, 39, 40, 52, 55, 58, 61–63, 67, 68, 71, 76, 77, 79, 89, 93, 95]
	Delays	6.8 (5)	
	Provider availability	8.1 (6)	
	Consultation time	6.8 (5)	
	Service availability	6.8 (5)	
	Referral	11.0 (8)	
Accessibility		14.9 (11)	[33, 36, 37, 54, 60, 61, 65, 67, 69, 77, 79]
	Environment, parking	9.4 (7)	
	Transport	5.4 (4)	
	Professional practice location	2.7 (2)	
	Lodgings	1.4 (1)	
Affordability		23.0 (17)	[19, 32, 36, 39, 47, 49, 55, 57, 61, 69, 74, 75, 83, 87, 96, 98, 103]
	Medical fees	5.4 (4)	
	Health insurance	10.8 (8)	
	Prescription fees	4.1 (3)	
	Cost of illness, economic	4.1 (3)	
	Affordability, general	5.4 (4)	
Accommodation		25.7 (19)	[32–34, 37, 52, 59, 60, 63, 64, 70, 71, 75, 77, 80, 87, 89, 90, 92, 101]
	Appointments and scheduling	4.1 (3)	
	Wait times	9.5 (7)	
	Out of hours care	6.8 (5)	
	Continuity of care	10.8 (8)	
	Provider contact	4.1 (3)	
	Accommodation, general	2.7 (2)	
Acceptability		75.7 (56)	[18, 19, 32, 34, 36–40, 42, 45, 47, 50–52, 54–57, 59–65, 67, 68, 70, 71, 73–80, 84–94, 96, 97, 99–103]
	Healthcare disparity, demographic	44.6 (33)	
	Decisional involvement	16.2 (12)	
	Health communication	27.0 (20)	
	Professional-patient relations (interpersonal skills)	17.6 (13)	
	Choice of professional	2.7 (2)	
	Clinical competence (technical skills)	8.2 (6)	
	Patient motivation or willingness to accept care	5.4 (4)	
Other barriers to optimal outpatient services		51.4 (38)	[32, 33, 35–37, 41, 43–48, 50, 52, 53, 55, 56, 58–61, 63, 65–67, 69, 71–73, 76, 77, 79–82, 84, 89, 93]
	Need assessment, undetected or untreated issues	25.7 (19)	
	Service amenities	12.2 (9)	
	Consumer information	32.4 (24)	
	Patient care team, coordination and medical record	9.5 (7)	
	Self care	5.4 (4)	
	Medical errors	2.7 (2)	

A total of 56 studies measured an acceptability barrier (75.7 %) and this was the most common barrier assessed. Within this domain, a total of 33 studies (44.6 %) reported patient demographics as a potential acceptability barrier to outpatient specialist care. It is important to note that demographic characteristics also served as moderator variables for other barriers. For example, male gender and lower income were associated with decreasing continuity of specialist care [101]. A total of 38 studies (51.4 %) examined other barriers (i.e., outside the model of fit) to optimal specialist care, including undetected or untreated physical or emotional issues and significant levels of unmet needs.

### Objective 2: Common and unique barriers experienced by patients with chronic diseases

Twenty three specific barriers were considered to be common across chronic diseases (Table 4) and ten were considered unique (Table 5). It is important to consider the number of studies reporting each of these barriers, particularly as the volume of articles differed between oncology and other chronic diseases. For example, sixteen oncology-specific studies reported communication with health professionals as an acceptability barrier [34, 36, 37, 40, 42, 51, 59, 60, 71, 76, 77, 80, 86, 91, 92, 94], whereas only four studies reported a similar barrier within any of the other eight diseases of interest [32, 56, 64, 93].

**Table 4:** Common barriers to specialist outpatient care by chronic condition and number of corresponding studies

Barrier	Reported in relation to:										Number of studies	
	CAN	AST	DEP	DIA	ISC	COP	ART	OST	STR	Total #	Oncology only (n = 53)	Other disease (n = 21)
Acceptability												
Decisional involvement	✓	✓	✓	✓	✓	✓	✓			7	9	3
Healthcare disparity by patient demographics	✓	✓	✓	✓	✓	✓	✓	✓	✓	9	22	11
Health communication	✓	✓	✓	✓	✓	✓	✓			7	16	4
Professional-patient relations	✓	✓	✓	✓	✓					5	12	1
Accessibility												
Parking	✓	✓	✓	✓	✓	✓	✓			7	6	1
Professional practice location	✓	✓	✓	✓	✓	✓	✓			7	2	1
Transport	✓	✓	✓	✓	✓	✓	✓			7	3	1
Accommodation												
Appointments and scheduling	✓	✓	✓	✓	✓	✓	✓			7	2	1
Continuity of care	✓	✓	✓	✓	✓	✓	✓	✓		8	4	3
Out of hours care	✓	✓	✓	✓	✓	✓	✓			7	2	3
Provider contact	✓	✓	✓	✓	✓	✓	✓			7	1	2
Wait times	✓	✓	✓	✓	✓	✓	✓			7	6	1
Affordability												
General affordability	✓	✓	✓	✓	✓	✓	✓	✓		8	2	2
Health insurance	✓	✓	✓	✓	✓	✓	✓	✓		8	5	3
Medical fees	✓	✓	✓	✓	✓	✓	✓			7	2	2
Prescription fees	✓	✓	✓	✓	✓	✓	✓			7	0	3
Availability												
Delays	✓	✓	✓	✓	✓	✓	✓			7	3	2
Service availability	✓	✓	✓	✓	✓	✓	✓			7	4	1
Optimal care												
Consumer information	✓	✓	✓	✓	✓	✓	✓			7	21	3
Medical errors	✓	✓	✓	✓	✓	✓	✓			7	1	1
Patient care team, coordination	✓	✓	✓	✓	✓	✓	✓			7	4	3
Self care	✓	✓	✓	✓	✓	✓	✓			7	3	1
Service amenities	✓	✓	✓	✓	✓	✓	✓			7	8	1

*CAN* Cancer, *AST* Asthma, *DEP* Depression, *DIA* Diabetes, *ISC* Ischaemic heart disease, *COP* Chronic obstructive pulmonary disorder, *ART* Arthritis, *OST* Osteoporosis, *STR* Stroke

**Table 5:** Unique barriers to specialist outpatient care by chronic condition and number of corresponding studies

Barrier	Reported in relation to:										Number of studies	
	CAN	AST	DEP	DIA	ISC	COP	ART	OST	STR	Total #	Oncology only (n = 53)	Other disease (n = 21)
Acceptability												
Choice of professional	✓									1	2	0
Clinical competence (technical skills)	✓									1	6	0
Patient factor	✓		✓							2	3	1
Accessibility												
Lodgings	✓									1	1	0
Accommodation												
General	✓									1	2	0
Affordability												
Cost of illness, economic	✓					✓				2	2	1
Availability												
Consultation time	✓									1	5	0
Provider availability	✓									1	6	0
Referral	✓		✓							2	6	1
Optimal care												
Inadequate need assessment	✓		✓							2	17	2

*CAN* Cancer, *AST* Asthma, *DEP* Depression, *DIA* Diabetes, *ISC* Ischaemic heart disease, *COP* Chronic obstructive pulmonary disorder, *ART* Arthritis, *OST* Osteoporosis, *STR* Stroke

### Common barriers

Within each domain, several barriers were common across chronic diseases (Table 4). As the most frequently described barrier to outpatient care, difference in service use, levels of need, or satisfaction according to demographic characteristics were reported across all diseases of interest [18, 19, 34, 38, 41, 45, 47, 51, 52, 54, 55, 57, 61, 64, 65, 68, 74, 75, 79, 84, 85, 87–89, 91, 92, 96, 97, 99–103]. Additional barriers resulting from sub-optimal interactions with healthcare teams or non-patient focussed health service organization were commonly reported.

Common barriers resulting from health service organization or physical structure included: waitlists and appointments delays [32, 33]; poor service availability [33, 52, 55, 58, 63]; difficulties with parking [33, 36, 37, 60, 65, 77, 79]; poor transport options [33, 36, 61, 69]; distance to the outpatient clinic [33, 54, 67]; inability to meet medical fees [32, 47, 49, 61] or prescription costs [32, 49, 83]; inadequate health insurance coverage [19, 39, 47, 55, 75, 83, 87, 98]; and poor service amenities [33, 37, 46, 60, 65, 71, 77, 79, 89].

Common barriers resulting from sub-optimal interactions with healthcare teams included: decisional involvement [32, 40, 50, 51, 56, 59, 60, 64, 67, 92, 71, 80]; communication with health professionals [32, 34, 36, 37, 40, 42, 51, 56, 59, 60, 64, 71, 76, 77, 80, 86, 91–94]; relations with health professionals [37, 38, 40, 42, 51, 59, 60, 64, 65, 71, 77, 80, 89]; inadequate information provision [32, 33, 36, 37, 45–47, 50, 52, 53, 55, 56, 59–61, 63, 65, 67, 69, 71, 77, 81, 82, 89]; poor coordination of care and information within the care team [32, 33, 43, 48, 60, 77, 80]; limited support for self-care practices [32, 59, 76, 82]; and medical errors [32, 77].

### Unique barriers

Ten barriers were considered unique and were predominately reported in oncology and depression samples (Table 5). Unique barriers to oncology care included access to or information on accommodation for those who were required to travel for treatment [69]; inadequate consultation time [34, 37, 40, 76, 79]; poor provider availability [37, 39, 55, 89, 95, 79]; professionals’ technical skills or clinical competence [37, 40, 42, 76, 79, 89]; and option to choose their healthcare professional [39, 63]. Cost of illness was reported as a barrier by both oncology and COPD patients [36, 49, 69].

Studies examining oncology, depression or the comorbid relationship between these diseases also reported: poor referral practice [52, 61, 62, 67, 71, 76, 93, 95]; inadequate need assessment [35, 41, 43–45, 47, 50, 52, 53, 59, 65–67, 72, 76, 80, 81, 84, 93]; and patient factors, such as motivation and willingness to accept care, as barriers to outpatient care [39, 62, 63, 76]. For example, patients’ level of perceived need was significantly associated with outpatient mental health service use [62]. Similarly, adult cancer survivors did not seek care if they felt they were in good health [63] or did not perceive the services were relevant to those in remission [39].

### Key themes and implications for health services

Results from this review suggest there are a wide range of barriers experienced by chronic disease outpatients. Following thematic analysis and synthesizing the results of the 74 reviewed studies, eight key themes according to the scope, frequency and commonality of barriers were found and are summarized below. Themes 1 through 6 are based on recurrent findings across individual studies. Themes 7 and 8 are reflections on the overall state of the evidence relating to barriers to specialist outpatient care. Health service or research implications for each of these themes can be found in Table 6 and provides a set of possible approaches for improving equity to high-quality specialist services.

**Table 6:** Summary of key themes and implications for health services and research

Summarized themes	Relation to study objective	Health service or research implications
Demographic characteristics create or exacerbate barriers	Frequent barrier	Improve breadth of patient participation and health literacy to reduce disparities
		Assess the degree to which services are culturally competent
		Target disadvantaged groups with additional supportive services
Availability barriers exist at first point of contact	Common barrier	Provide explanations for and estimates of delays
Service structures create accommodation and accessibility barriers	Common barrier	Improve appointment scheduling systems:
		- record individual preferences for date and time
		- coordinate all required appointments at the facility on one day
		-convenient rescheduling process
		Decrease wait-times
		Incorporate notification system for estimated wait-times
Continuity and coordination of care poses barriers	Common barrier	Improve content and access to medical records:
		-systematic data collection for accuracy and completeness
		ability to record additional patient concerns
		-notification or alerts when test results are available
		-centralized progress summaries for multiple service providers
Decisional involvement and information provision impacts acceptability of care	Common barrier	Provide personalized information to patients
		Provide ongoing opportunities to review progress and concerns
		Provide access to additional information sources
		Provide communication training for providers
		Consider and discuss individual patient preferences for decisional involvement
Need assessment and referral processes for cancer and/or depression can be improved	Unique barrier	Conduct systematic, comprehensive and routine screening of patients’ needs
		Refer automatically to support services
		Inform health professionals of additional services available
Barriers can be described in additional detail	Scope of barrier	Deconstruct barriers to design more targeted initiatives for improving access
Evidence on barriers to non-oncology services is limited	Volume of articles	Barriers reported within clusters of conditions mask differences across groups
		Conduct more studies in non-oncology patient groups

#### Theme 1: Patient demographic characteristics frequently create or exacerbate barriers

Of the reviewed articles, the most frequently reported barrier to care examined was acceptability. This was primarily due to the focus on patients’ demographic characteristics as both a barrier to receiving optimal care and as a critical factor in mediating the magnitude of barriers experienced. Examples include examining disparities according to race [18, 19, 91, 102]; education [61, 71]; age [65, 71, 89, 92]; gender [51, 52, 65]; presence of comorbidities, disease severity or reduced health status [89, 92, 103]; and socioeconomic groupings [74, 96, 101]. For example, using the population-based Nord-Tondelag Health Survey (HUNT-3), Vikum et al. explored the differential use of healthcare according to patients’ education levels and household income [74]. The large sample (n = 44,755) included patients who self-reported suffering from one or more of 18 chronic diseases including: cancer; diabetes; respiratory illness, such as COPD and asthma; musculoskeletal disorders, such as arthritis and osteoporosis; and stroke. Overall, the need for all services was greatest for those in lower income groups and a positive significant relationship exists between both income and education levels and the use of outpatient specialist services, with the exception of males aged 20–39 years.

Socioeconomic status was a common demographic variable of interest and, as one would expect, was related to an individual’s ability and willingness to pay for services. Approximately 17 articles explored a barrier resulting from the cost of healthcare [19, 32, 36, 39, 47, 49, 55, 57, 61, 69, 74, 75, 83, 87, 96, 98, 103]. These articles were conducted in a variety of healthcare systems, including those with publically-funded healthcare schemes designed to encourage universal access to services. This suggests that patients still must contend with several other sources of financial strain resulting from the need to access healthcare, such as lost-income or out-of-pocket spending. While this review cannot describe the differences in the barriers experienced according to the funding structures of different OECD countries, results suggest that the affordability of healthcare and the disparities that result are still a source of considerable patient concern. This is supported by previous research [32].

#### Theme 2: Common availability barriers exist at first point of contact with health services

A range of availability barriers, such as delays to treatment, are considerable concerns for patients when first accessing services. Across chronic disease groups, barriers exist at first point of contact with a particular service and include delays to receiving care and limited provider availability. Delays to receiving care were reported across multiple chronic diseases and were frequently experienced within several high-income countries. Within Canada, Australia, New Zealand and the United Kingdom, the majority of patients who experienced recent ill health did not receive specialist care within 4 weeks [32]. Acceptable wait periods to receive treatment or surgical interventions have been established within national guidelines, mainly to optimize patient outcomes [68]. However, wait times also pose a significant concern from a patient perspective. Paul et al. report approximately 52 % of Australian radiotherapy outpatients experienced some level of concern regarding delays in treatment [68]. Patients expect that care, particularly for recently-diagnosed prevalent chronic disease (e.g., diabetes), should be received in a timely manner and ideally within 14 days from receiving a referral [33].

#### Theme 3: Health service structure and organization create common accommodation and accessibility barriers

Synthesized results suggest patients continue to experience barriers over the course of their interaction with health services and patients’ preferences are not accommodated within health service organization. These barriers include non-clinical aspects of the service’s physical structure, such as difficulties with parking. For patients who must access services for treatment, such as intravenous chemotherapy, parking remains a major issue [33, 36, 37, 60, 65, 77, 79]. For example, within a study of cancer patients in the United Kingdom, parking was rated as the least met need despite being rated as a highly salient [65]. Non-clinical accommodation barriers were also experienced as a result of the service organizational structure. This was predominately reported by patients’ dissatisfaction with appointment scheduling [71], appointment wait times, inability to contact the clinic or professionals [64], or limited availability of out of hours care [59].

Up to 60 % of oncology outpatients reported that waiting times of more than 15 min contributed to poor experiences within health services [59] and lengthy wait times accounted for a third of all patient-reported experiences of poor care [77]. This represents a potential area of improvement as wait times are highly salient to patient experiences [70] and patients who experienced lengthy wait times were more likely to report significantly lower levels of satisfaction and perceive shorter consultation times [34]. Inadequate consultation times were also reported as a barrier to oncology outpatient care. Studies using the EORTC OUTPATSAT35 report both physician punctuality and the amount of physician time devoted to the patient were the worst performing subscales and received scores below 70 [79, 89]. Patients identified sufficient time to review all questions regarding disease and treatment and having their physician’s complete attention as being very important when receiving a diagnosis [40].

#### Theme 4: Common patient barriers are reported as a result of poor coordination of care

In 2008, the Commonwealth Fund International Health Policy Survey of Sicker Adults reported a considerable proportion of patients believed their medical care was inefficient or wasteful (rates range from 27-46 %) [32]. This negative perception of care may be a result of poor clinical competence of health professionals, lack of continuity of care, poor coordination of the patient care team, and medical errors.

Continuity and coordination has been associated with improved patient care and is frequently assessed according to patients’ access to a usual source of care. For example, having a usual care provider was associated with improved screening and use of outpatient services for diabetic patients [101] and treatment of depression for patients with comorbid diseases [75]. Advanced lung cancer patients identified as having experienced poor continuity of care were more likely to have unmet supportive care needs across domains such as health information and psychological needs [80]. In addition to improved patient care and outcomes, studies consistently identify continuity of care as essential to patients’ experiences of care. In a study of elderly patients’ priorities for health service delivery, patients rated continuity as the most important aspect of care with approximately 94 % indicating it was extremely important to see the same physician at every appointment [33]. Similarly, three studies of young adults found the majority of patients prefer follow-up care to be delivered by their treating physician and service [34, 52, 63].

In a study of patients’ perceptions of outpatient care in eight Commonwealth countries, poor continuity or availability of information within the healthcare team was reported [32]. This included: non-availability of medical records or test results at time of scheduled appointment; unnecessary duplication of tests; and poor information exchange between general practitioners and specialists. Discrepancies in medical records were also reported by reviewed studies. For example, a review of oncology medical records revealed only 49 % of symptoms were documented and patient-identified issues, such as difficulties with mobility or maintaining activities of daily life, were frequently omitted [43]. Similar discrepancies between patient-identified symptoms and documentation have been reported for patients with chronic heart disease [48].

#### Theme 5: Aspects of the patient-physician relationship can negatively impact the acceptability of care

Barriers in the patient-physician interaction arose when examining decisional involvement, communication, and information provision. Approximately 70 % of oncology patients reported there was a difference between the ideal and actual physician relationships [51]. Of this, approximately 32 % of patients reported poor decisional involvement and 28.5 % did not feel encouraged by their physician. Across the 13 domains of the PASQOC survey, co-management and shared decision making had the second highest problem frequency (30 %) with a large proportion of oncology patients indicating they did not make the treatment decision (47 %) and were not effectively informed on the probability or management of side-effects (49 % and 38 %, respectively) and changes to daily life (37 %) [59]. Furthermore, 34 % did not feel as if they were treated as an expert on their body.

Across multiple diseases, considerable gaps in patient-provider communication were reported and included: patient preferences and goals for treatment are not discussed (26-50 %); patients are rarely or only sometimes encouraged to ask questions (24-38 %); and are rarely or only sometimes told about treatment options and involved in decisions (12-31 %) [32]. Within COPD and chronic heart failure (CHF) outpatients, only within 5.9 % of COPD group and 3.9 % of CHF group did both patient and physician report discussing preferences for life-sustaining treatment [56].

Information on the impact of treatment and potential trade-offs between quality and prolongation of life is typically communicated by treating physicians. Within study results, patients identify information content as the most important aspect of a clinical appointment [40]. Patients attribute high importance to being informed on the best treatment options and being aware of all treatment options [40]. Additionally, patients would like to be aware of prognoses, treatment results and be provided with information on their personal situation [71]. Within oncology outpatients, patients identified a lack of information on changes in relationships, sexual activity, or emotions was an area of improvement [67]. Poor communication and information provision for family and close others was also reported within the review as an area of relatively lower quality [51, 71].

#### Theme 6: Inadequate need assessment and referral practices are unique barriers experienced in relation to few chronic diseases

Patients with cancer and/or depression diagnoses report unmet needs and referral processes as a barrier to optimal outpatient care. For patients diagnosed with depression and/or cancer, synthesized study results suggest that health professionals do not consistently identify psychological or physical symptoms. For example, within outpatient oncology clinics only 49 % of patients with major depressive disorder (MDD) reported speaking to a health care professional about feeling depressed (albeit this study did not distinguish between a primary care provider or oncologist for this stage of screening); 36 % reported receiving any subsequent treatment or a referral to a specialist mental health service; and in total authors estimated up to 85 % of patients did not receive appropriate specialised treatment for MDD [93]. Slightly higher rates of treatment for moderate to severe symptoms of depression (61.9 %) and anxiety (60.6 %) were reported in a sample of several outpatient clinics specializing in cancer and chronic disease care, but this remained sub-optimal [62].

Referral processes was also reported by patients as a critical gap in the provision of outpatient care. Automatic referral to a social worker for financial, emotional, and organizational concerns was rated as important by young adults currently receiving or having completed oncology treatment [52]. Only one in two patients are referred to a social workers due to resource limitations [95]. Referral to and availability of services such as nutritional counselling, physical therapy, support groups and rehabilitation were also reported by cancer outpatients as highly important to optimal outpatient care [61]. Only one in two patients reported using such supportive services and patients’ lack of knowledge of these services (22.4 %) or lack of physician referral (23 %) was reported as the main reasons for underuse. Referral was the strongest predictor of recent mental health treatment (OR = 7.91) as compared to variables such as appointment frequency, perceived need, and prior use [62].

#### Theme 7: To provide more practice-ready evidence, barriers to outpatient specialist care should be described in additional detail

A total of 30 distinct barriers were reported within the reviewed papers. Consideration of the scope of these barriers using MeSH terms suggests that it is important to go beyond the overarching barriers such as the volume or affordability of available services. For example, affordability was explored in 17 papers and described four distinct forms of affordability barriers experienced by chronic disease outpatients: inadequate health insurance coverage; inability to meet the costs of medical services; inability to afford prescriptions; and the cost of illness, such as lost income for those who are unable to work. Each of these barriers requires a different type of solution suggesting it is important to have detail about the barrier in order to take appropriate action.

#### Theme 8: This review found little evidence on barriers to non-oncology services

There is a wealth of information on the barriers to outpatient oncology care, but barriers experienced by other chronic illnesses are less understood. A total of 59 articles described a barrier to oncology services. Comparatively, few studies (15 of 74) focused on other chronic illnesses and typically analysed barriers within a heterogeneous sample of diseases. However, it is important to note that this review may not have captured the barriers experienced by some chronic disease groups, such as people with osteoporosis, because of the limited focus on specialist services. It is possible that these groups are adequately managed within primary care settings and do not frequently require access to specialist care.

### Limitations

It is possible that publication bias affected the results of this systematic review, whereby articles with significant results are more likely to be accepted in peer-review journals. By accessing only peer-reviewed studies it is possible study results over-estimates the barriers experienced by chronic disease outpatients. Grey literature or qualitative articles may have provided additional or alternative views of access to care. Additionally, most studies employed a cross-sectional survey design which may not have provided a longitudinal view of patients’ ongoing experience with care. However, given the large number of articles reviewed with a range of patient samples, results are inclusive of several areas of care such as diagnosis, treatment decisions, and ongoing patient needs.

Barriers were classified according to Medical Subject Headings and grouped according to definitions proposed within Penchansky and Thomas’ model of fit [14]. This required some subjectivity on behalf of the research team and the team generated a sixth barrier relating to dimensions of patient-centered care. While coding processes and data extraction was pilot-tested and agreement verified, several assumptions regarding these classifications were made. For example, specific barriers such as health communication and professional-patient relations are intertwined concepts necessary for a patient-centered approach to care. There are additional access frameworks, such as that proposed by Donabedian [104], and Andersen and Aday [105], which could have been applied within this review. Debate on the value of each framework is presented elsewhere [12], and research to evaluate the degree to which these models are inclusive of emerging quality of care dimensions would be valuable.

Subjective judgements were required when reviewing the results in order to generate thematic concepts. While this is an inherent limitation of an interpretive review, this allowed authors to provide a more concise summary of the recurrent barriers reported by a large volume of articles employing a range of measurement approaches in markedly different patient groups. Themes were generated according to well-established qualitative methods [106].

## Conclusions

Overall, patients with prevalent chronic diseases experience thirty three specific barriers to outpatient care across six accessibility domains. This includes additional patient-centered care dimensions such as self-care, consumer information provision, and need assessment. By focusing on prevalent chronic diseases within outpatient specialist settings, this systematic review describes the scope and frequency of common and unique barriers to care and synthesizes this into a concise list of potential quality improvement initiatives.

Results from this review suggest that in order to design targeted initiatives, it is important characterize barriers in detail and to explore possible barriers in the delivery of patient-centered care. In examining the common barriers, four themes were recurrent across chronic disease groups. First, at initial contact with a health care service, individuals experience delays to first appointment or treatment and causes considerable patient concern. Second, patients report health services are not organized or sufficiently flexible to accommodate scheduling preferences, and the physical structure of the clinic limits accessibility. Third, poor continuity of care and information transfer in the healthcare team was perceived to negatively impact the quality of care received. Fourth, inadequate information provision and a lack of involvement in treatment decisions were reported by multiple chronic disease groups. Given these themes were recurrent across chronic disease groups, system-wide initiatives targeting these gaps in the quality of care are appropriate and should be prioritized. Health services may consider improvements in: appointment scheduling systems; content of and access to medical records across health professionals; and timely provision of personalized information with multiple opportunities to review patient concerns.

In examining the unique barriers experienced by only a few chronic disease groups, need assessment practices and referral processes were seen as sub-optimal by individuals diagnosed with cancer and/or depression. Health services may consider evaluating current screening practices to ensure need assessments are: routinely and systematically conducted; sufficiently flexible to document salient needs that may be outside the scope of physical or emotional concerns, such as psychosocial or spirituality needs; and provide instruction and a process to address a detected need, such as an automatic referral pathway. Results from this study suggest these initiatives may best targeted within oncology or mental health services.

## Additional file

Additional file 1:
**Example of the electronic search strategy, including Medline database search terms and limits.**
