# Supplementary material for: A systematic review of barriers to optimal outpatient specialist services for individuals with prevalent chronic diseases: what are the unique and common barriers experienced by patients in high income countries?
Source: Int J Equity Health. 2015 Jun 9;14:52. doi: 10.1186/s12939-015-0179-6 (PMC4464126; doi:10.1186/s12939-015-0179-6)
Supplement: Additional file 1: — Example of the electronic search strategy, including Medline database search terms and limits. [file 12939_2015_179_MOESM1_ESM.docx]

***Additional file 1: Example of the electronic search strategy, including Medline database search terms and limits***

| **Information source** | **Search terms and associated Boolean operator** | **Limits** |
| --- | --- | --- |
| Medline | 1. Neoplasms or Chronic Disease (subject headings),  **AND**  2. Ambulatory Care Facilities or Ambulatory Care or Outpatient (subject headings),  **NOT**  3. Assisted Living Facilities or Homes for the Aged or Housing for the Elderly or Palliative Care or Terminal Care or Urgent Care or Emergency Medical Services (subject headings),  **NOT**  4. Child* or Infant or Adolescent or Paediatric (all fields) | 1. All adults (19 plus years)  2. Humans  3. English language  4. Year = 2002 to Current (April 15^th^ 2012 or May 16^th^ 2014) |
